# Evidence for In Utero Hematogenous Transmission of Group B β-hemolytic Streptococcus

**DOI:** 10.1155/S1064744994000621

**Published:** 1994

**Authors:** Kathleen Robischon, Marvin S. Amstey

**Affiliations:** Department of Obstetrics and Gynecology University of Rochester School of Medicine and Dentistry Highland Hospital 1000 South Avenue Rochester NY 14620 USA

## Abstract

*Background:* The presumed ascending route of group B β-hemolytic streptococcus (GBS) infection from the colonized maternal genital tract is well accepted. This case report proposes a hematogenous, selective infection of one unruptured amniotic sac over the other ruptured amniotic sac in a twin gestation in a patient with known GBS vaginal colonization.

*Case:* This is a case report of GBS sepsis in twin B with intact membranes. Twin A, with 28 h of ruptured membranes, failed to show any signs of infection. The pathology of the placenta confirmed chorioamnionitis in twin B and the absence of infection in twin A.

*Conclusion:* The presence of culture-positive GBS sepsis in the twin with the unruptured amniotic sac, as well as the absence of GBS infection in the twin with the ruptured sac, suggests an alternative means of infection for GBS infection, such as hematogenous transplacental transmission.


Infectious Diseases in Obstetrics and Gynecology 2:184–185 (1994)
© 1994 Wiley-Liss, Inc.

### Evidence for In Utero Hematogenous Transmission of Group B β-Hemolytic Streptococcus

**Kathleen Robischon and Marvin S. Amstey**

*Department of Obstetrics and Gynecology, University of Rochester School of Medicine and Dentistry, and Highland Hospital, Rochester, NY*

Abstract

**Background:** The presumed ascending route of group B β-hemolytic streptococcus (GBS) infection from the colonized maternal genital tract is well accepted. This case report proposes a hematogenous, selective infection of one unruptured amniotic sac over the other ruptured amniotic sac in a twin gestation in a patient with known GBS vaginal colonization.

**Case:** This is a case report of GBS sepsis in twin B with intact membranes. Twin A, with 28 h of ruptured membranes, failed to show any signs of infection. The pathology of the placenta confirmed chorioamnionitis in twin B and the absence of infection in twin A.

**Conclusion:** The presence of culture-positive GBS sepsis in the twin with the unruptured amniotic sac, as well as the absence of GBS infection in the twin with the ruptured sac, suggests an alternative means of infection for GBS infection, such as hematogenous transplacental transmission.
© 1994 Wiley-Liss, Inc.

Key words

Pregnancy, maternal colonization, chorioamnionitis, twin gestation, group B streptococcus

Group B β-hemolytic streptococcus (GBS) in the fetus or neonate is believed to be transmitted by an ascending route from the colonized maternal genital tract, including the vagina, anorectal area, and genitourinary tract. The fetus acquires infection from aspiration or swallowing or through mucous membranes.[1] Thus, prolonged rupture of membranes is a significant risk factor for transmission of this agent. Other risk factors for neonatal infection include preterm delivery, prolonged labor, intrapartum fever, and chorioamnionitis.[2] This report documents a case of GBS sepsis in twin B, whose membranes were unruptured prior to delivery in a mother with a positive cervical culture for GBS. Twin A, who had ruptured membranes for 28 h, was asymptomatic with no evidence of infection.

### CASE REPORT

The patient was a 30-year-old gravida 1 at 34 weeks with a known twin gestation. She presented 1 h after rupture of membranes to labor and delivery. The amniotic fluid was noted to be clear. There was no history of vaginal bleeding or contractions. Her history was complicated by primary infertility resulting in the current pregnancy having been conceived on clomiphene and gonadotropins. The patient underwent selective reduction of quadruplets to twins at 14 weeks gestation. Otherwise the pregnancy had been uncomplicated, and the patient had been treated only with modified bed rest from 28 weeks gestation.

A sterile speculum examination confirmed rupture of the membranes, and fluid from the vaginal pool was obtained for culture and estimation of

Address correspondence/reprint requests to Dr. Marvin S. Amstey, Department of Obstetrics and Gynecology, University of Rochester School of Medicine and Dentistry, Highland Hospital, 1000 South Avenue, Rochester, NY 14620.

Received June 15, 1994
Accepted September 6, 1994

phosphatidylglycerol. She was afebrile with a normal white blood cell (WBC) count, differential, and hematocrit. There was no evidence of chorioamnionitis. Approximately 12 h after admission, she began feeling contractions and had an episode of shaking chills. Her temperature rose to 38.1°C, and her WBC count increased from 9,000 to 12,000. A cervical examination revealed dilatation to 4 cm with contractions every 4 min. Twin A was in a vertex compound presentation in which the hand was presenting alongside the head. Twin B was in the frank breech presentation. Ampicillin was started at 2 g q 6 h. Both fetal heart rate patterns were reassuring.

The patient progressed to 7 cm dilation at which time twin B (unruptured) had an episode of significant bradycardia which led to an immediate cesarean section. Twin A, a male weighing 2,450 g, was delivered with a strong cry, vigorous movement, and Apgar scores of 7 and 9 at 1 and 5 min, respectively. Twin B, a female weighing 1,870 g, was delivered from an intact amniotic sac with Apgar scores of 1, 5, and 6 at 1, 5, and 10 min, respectively. This infant required full resuscitation and was given ampicillin and gentamicin directly into the umbilical vein in the delivery room. Twin B (unruptured) had blood cultures that were positive for GBS in <24 h. Twin A (ruptured) had all negative cultures; however, both infants were treated for a full 10 days with antibiotics. The mother, whose amniotic fluid culture grew "light GBS," was treated with ampicillin/sulbactam after she developed signs of endometritis. She was discharged home after 5 days; twin A was discharged home after 10 days; and twin B was discharged home after 15 days.

The pathology report demonstrated a dichorionic-diamnionic placenta. The amniotic sac for twin B showed acute chorioamnionitis while the amniotic sac for twin A showed only a few small infarcts, a hemangioma, but no evidence of chorioamnionitis.

### DISCUSSION

The obvious sepsis in twin B (unruptured) and the absence of sepsis or any manifestation of GBS infection in twin A (ruptured) despite premature and prolonged rupture of the membranes go against our current understanding of the mechanism of GBS infection in the neonate. If ascending infection from maternal vaginal colonization with GBS to the fetus/neonate[3] were the only, or principal, means of infection, we would presume that twin A would be most susceptible. This would not explain how twin B, with intact membranes, was infected.

There are several case reports in the literature of hematogenous spread of bacteria and viruses in which selective infection of one gestational sac over the other may occur. For example, *Listeria* infection in a twin gestation at 27 weeks resulted in the stillbirth of one twin with disseminated disease. The other twin was relatively stable at birth but succumbed to *Listeria* meningitis at age 5 months.[4] Vogler et al.[5] demonstrated disseminated cytomegalovirus infection in a stillborn twin without any infection in the other twin of a dichorionic-diamnionic gestation. This case proposes the possible hematogenous transplacental transmission of GBS.

An alternative explanation could be a leak in the membranes of the unruptured twin that had sealed over prior to delivery, indicating ascending infection as a possibility. However, this would not explain why the twin with prolonged rupture of the membranes did not manifest any signs of infection. It appears that hematogenous transmission of GBS played a role in causing the pronounced infection in the unruptured twin.

If hematogenous transmission of GBS does occur, it could have a significant impact on the management of patients known to be GBS carriers. This possibility adds to the controversy over when to test for GBS and when to initiate antibiotic therapy, as well as the role for a GBS vaccine.

### REFERENCES

1. Pass M, Gray B, Dillon H: Puerperal and perinatal infection with group B streptococci. Am J Obstet Gynecol 143: 147, 1982.
2. Dillon H, Khare S, Gray B: Group B streptococci carriage and disease: A 6 year prospective study. J Pediatr 110:31, 1987.
3. Anthony B, Oxada D, Hobel C: Epidemiology of the group B streptococcal infections: Longitudinal observation in pregnancy. J Infect Dis 137:524, 1978.
4. Bigrigg A, Chissell S, Swingler G: Listeriosis in a twin pregnancy. Br J Hosp Med 45(3):171, 1991.
5. Vogler C, Kohl S, Rosenberg H: Cytomegalovirus infection and fetal death in a twin. J Reprod Med 38(2):142, 1993.
